# Ultralong Durability of Porous α‐Fe_2_O_3_ Nanofibers in Practical Li‐Ion Configuration with LiMn_2_O_4_ Cathode

**DOI:** 10.1002/advs.201500050

**Published:** 2015-03-30

**Authors:** Sundaramurthy Jayaraman, Vanchiappan Aravindan, Mani Ulaganathan, Wong Chui Ling, Seeram Ramakrishna, Srinivasan Madhavi

**Affiliations:** ^1^ Environmental and Water TechnologyCenter of InnovationNgee Ann PolytechnicSingapore599489; ^2^ Energy Research Institute @ NTU (ERI@N)Nanyang Technological UniversityResearch Techno Plaza50 Nanyang DriveSingapore637553; ^3^ Center for Nanofibers and NanotechnologyDepartment of Mechanical EngineeringNational University of SingaporeSingapore117576; ^4^ School of Materials Science and EngineeringNanyang Technological UniversitySingapore639798; ^5^ TUM‐CREATE1 Create way #10–02 CREATE TowerSingapore138602

**Keywords:** α‐Fe_2_O_3_ nanofiber anode, conversion anodes, full‐cell, irreversible capacity loss, Li‐ion battery

## Abstract

**Prelithiated, electrospun α‐Fe_2_O_3_ nanofibers** display an exceptional cycleability when it is paired with commercial LiMn_2_O_4_ cathode in full‐cell assembly. The performance of such α‐Fe_2_O_3_ nanofibers is mainly due to the presence of unique morphology with porous structure, appropriate mass balance, and working potential. Also, synthesis technique cannot be ruled out for the performance.

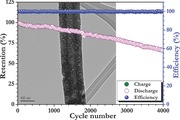

By virtue of their high reversible capacity, power capability and electrochemical stability make conversion or displacement type anode as prospective material for the construction of high power Li‐ion power packs compared to the insertion type anode, graphite.^1^ Though, graphitic anode is dominated in the commercial Li‐ion battery market, but the feasibility of using such electrodes are not possible in the high power requirements because of the poor rate capability and associated Li‐platting issue toward electric vehicle (EV) and hybrid electrical vehicle (HEV) application point of view.^2^ Alternatively, several insertion type anodes such as Li_4_Ti_5_O_12_, LiCrTiO_4_, anatase, and bronze phases of TiO_2_, TiNb_2_O_7_, TiP_2_O_7_, etc., are proposed as promising candidates to replace graphite.^1^, ^3^ Such alternate insertion hosts delivered much better electrochemical performance than graphitic anodes especially at high current rates, but the reversible capacity is highly limited compared to its counterpart (graphite). Therefore, much research activities are focused to develop either alloy or conversion type anodes for the fabrication of high power Li‐ion cells with high reversible capacity toward EV and HEV perspective.^4^ Unfortunately, the huge volume variation and unstable solid electrolyte interface formation (SEI) certainly offset the potential use in practical configuration compared to latter type electrodes, though Sony's Nexelion configuration is exceptional one (but it contains the Co as conversion type element).^5^, ^6^ Hence, the possibilities of using conversion type anodes for the construction of Li‐ion cells are highly warranted and research efforts on such materials are carried out in a full swing in the recent past.^7^, ^8^, ^9^, ^10^, ^11^

Poizot et al.^12^ first explored the possibility of using nanosized transition metal oxides as promising candidate for the construction of high power and high energy Li‐ion cells and the same concept has been extensively adopted for various binary and ternary metal oxides which undergo conversion mechanism.^1^ In addition, transition metal nitrides, sulfides, fluorides, chlorides, hydroxides, and carbonates have also been explored as anode for LIB applications under the similar conversion mechanism. Among the conversion anodes reported, Fe‐based oxides such as Fe_2_O_3_, Fe_3_O_4_, etc., are found appealing in terms of high reversible capacity, appreciable reduction potential (≈0.8 V vs. Li), easy synthesis protocol, natural abundance, low‐cost, and eco‐friendliness.^13^, ^14^, ^15^ In particular, Fe_2_O_3_ exhibits the theoretical capacity of ≈1007 mA h g^−1^ for the six electron reaction (Fe_2_O_3_ + 6 Li^+^ + 6e^−^ ↔ Fe^0^ + 3Li_2_O) and exhibits high reversibility as well. Irreversible capacity loss (ICL) remains an issue for both conversion and alloy type anodes while fabricating the full‐cell with conventional cathodes.^1^, ^6^ To keep everything in mind, we made an attempt to employ the scalable electrospinning technique to prepare the hematite phase preferably by 1D nanofibers with phase pure structure.^16^, ^17^ So far, to overcome the ICL issue, several pretreating procedures such as chemical lithiation,^8^ electrochemical lithiation of either single (anode)^8^ or both electrodes (anode and cathode)^9^ or taking excess loading of cathode^18^ or usage of sacrificial lithium salts in electrolyte^19^ or adopting stabilized lithium metal powder^20^ have been successfully attempted. Now, the prelithiation process is well matured and already been commercialized for the fabrication of Li‐ion capacitors.^21^ However, for the preliminary or lab scale studies, the electrochemical lithiation process toward either anode or cathode is sufficient and it can be easily transferred in to chemical lithiation technique during the mass production. Hence, the electrospun α‐Fe_2_O_3_ is pretreated by electrochemical lithiation and subsequently assembled in full‐cell configuration with commercial LiMn_2_O_4_ cathode. Before conducting the full‐cell assembly, mass loading between the electrodes are adjusted based on the electrochemical performance of the individual electrodes in half‐cell configuration under the same current rate. In addition, extensive structural and morphological studies are also performed and described in detail.

**Figure** **1** represents the structural and morphological features of the porous α‐Fe_2_O_3_ nanofibers prepared by well established electrospinning technique. The XRD reflections clearly indicate the formation of single phase α‐Fe_2_O_3_ and there is no evidence of secondary phase materials such as FeO or Fe_3_O_4_ etc., observed (Figure 1a). The lattice parameter values are calculated and found to be *a* = 5.033 (8) Å and *c* = 13.745 (3) Å with crystallite size value of ≈46 nm. The observed values are consistent with literature values (JCPDS Card No. 33–0664) and indexed according to the $R\bar{3}C$ space group. It is well known that the nanostructured materials with porous structure certainly translate much better electrochemical activity than conventional materials because of its more exposed surface area for the conversion reaction. The FE‐SEM pictures clearly revealed the presence of fiberous morphology with highly interconnected mat (Figure 1b). Average fiber diameter of ≈100 nm is noted which clearly seen from the FE‐SEM pictures (Figure 1c). The presence of porosity and smooth surface morphology is clearly supported from the TEM images with various magnifications (Figure 1d–g). The presence of such pores allows the penetration of electrolyte solution, which enables complete participation of the active material. As a consequence, high capacity will be resulted. Further, high resolution TEM pictures and selected area electron diffraction (SAED) pattern clearly revealed the crystallinity of the prepared porous hematite nanofibers (Figure S1, Supporting Information).

**Figure 1:**
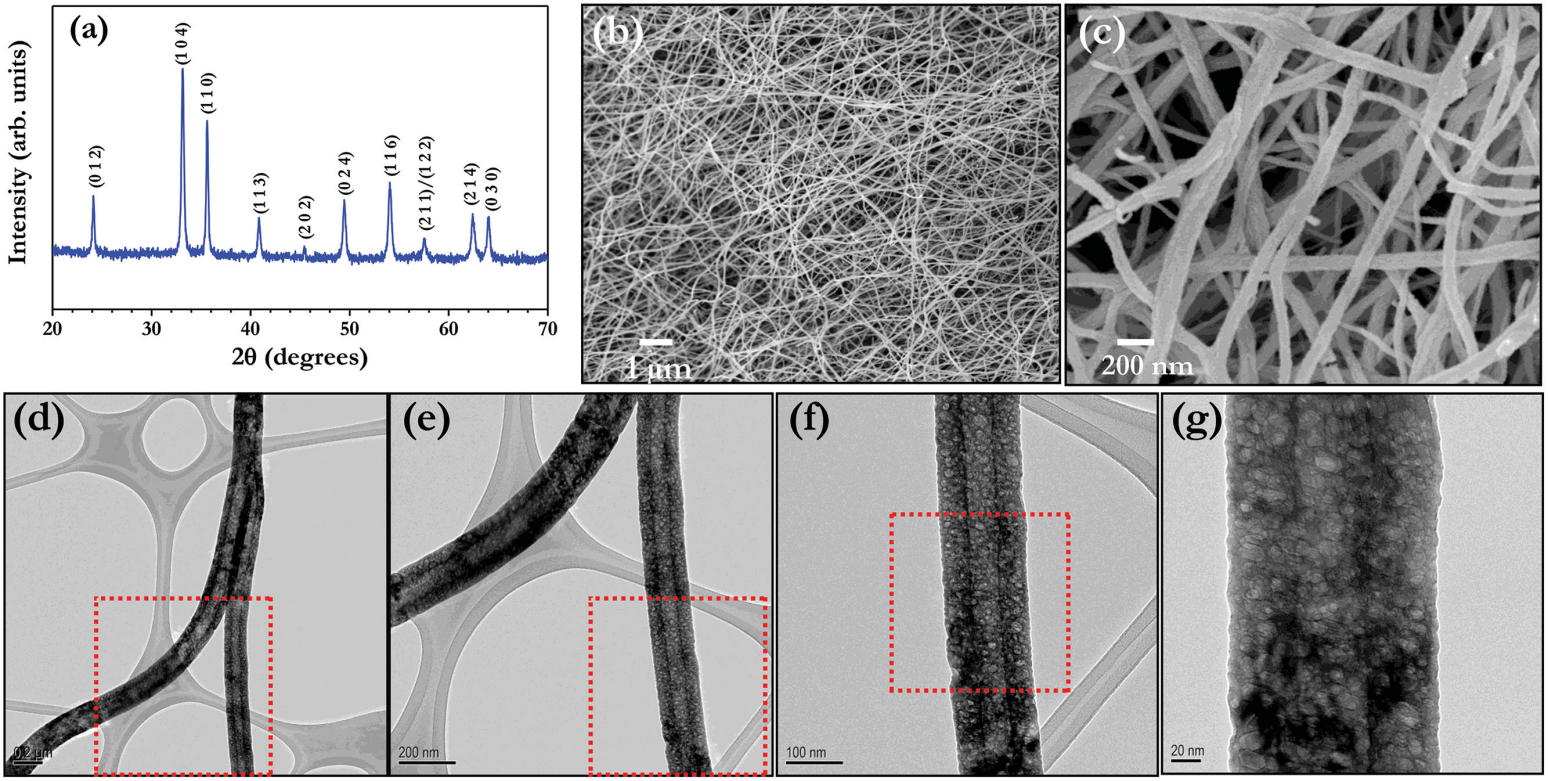
a) Powder X‐ray diffraction pattern of porous α‐Fe_2_O_3_ nanofibers, b,c) FE‐SEM images, and d–g) TEM pictures with different magnifications.

Li‐storage properties of the electrospun porous α‐Fe_2_O_3_ nanofibers were first evaluated in half‐cell assembly between 0.005–3 V vs. Li at 0.1 C rate (1 C is assumed to be 1000 mA g^−1^). Typical galvanostatic profile of Li/α‐Fe_2_O_3_ cell was given in **Figure** **2** along with the differential capacity profile. The discharge curve clearly showed the multiple reactions occurred in the first cycle. More clearly, at ≈1.6 V vs. Li, ≈0.5 mole of Li is inserted in to the hematite structure and leads to the formation of hexagonal Li_0.5_Fe_2_O_3_ phase.^15^ Upon continuous discharge, further intercalation of ≈1.23 mole of Li is noted in to the hexagonal phase at ≈1.08 V vs. Li which eventually results the formation of cubic Li_1.73_Fe_2_O_3_ phase. The formation of both hexagonal and cubic phase formation is occurred in an irreversible manner. The presence of long distinct plateau ≈0.9 V vs. Li corresponds to the destruction of the cubic Li_1.73_Fe_2_O_3_ phase, i.e., the formation metallic Fe^0^ particles and along with the amorphous Li_2_O.^15^ In addition to the aforesaid reaction, the decomposition of the organic electrolyte and subsequent formation of SEI layer cannot be ruled out. Generally, the SEI layer consists of insoluble inorganic by products such as Li_2_CO_3_, LiF, etc., and oligomeric films as well.^22^, ^23^, ^24^ As a consequence, the SEI formation consumes more amounts of Li in an irreversible manner, which leads to the high capacity than the theoretical predictions. The half‐cell delivered the capacity of ≈1748 and ≈1180 mA h g^−1^ at 0.1 C rate for first discharge and charge, respectively. The ICL of ≈568 mA h g^−1^ is noted which corresponds to the ≈62% of reversibility in the first cycle and consistent with the literature reports as well.^1^, ^15^, ^25^ However, the observed reversible capacity, ≈1180 mA h g^−1^ is higher than the theoretical limitations, which is mainly because of the non‐Faradic contribution, i.e., pseudocapacitance from the interfacial reaction across the metal/Li_2_O phase boundary.^25^ Similar kind of higher reversibility is also observed by the other researchers for the hematite system.^25^, ^26^, ^27^ The differential capacity profile clearly corroborates the aforesaid reaction described based on the galvanostatic studies. Plot of capacity vs. cycle number is given in Figure 2b. Apparently, a notable cycleability is observed for the case of electrospun nanofibers compared to the previous reports on such fibers.^25^, ^26^ It is worth to mention that the coloumbic efficiency of the cell tends to increase upon cycling and observed over 96% efficiency after 20 cycles.

**Figure 2:**
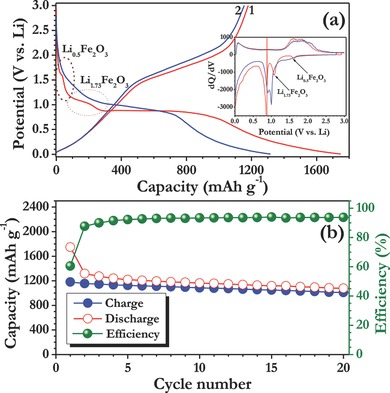
a) Typical galvanostatic charge–discharge curves of hematite nanofiber in half‐cell assembly (Li/α‐Fe_2_O_3_) between 0.005–3 V vs. Li at constant current density of 100 mA g^−1^. (Inset) Differential capacity profile for the given two cycles. Li‐insertion in to hematite structure is marked with Li intake. b) Plot of capacity vs. cycle number with coloumbic efficiency.

Our main intention is to develop long‐lasting Li‐ion cell using conversion type anode toward the EV and HEV application point of view with high energy and power capabilities. In this line, an attempt has been made to employ electrospun α‐Fe_2_O_3_ nanofibers as anode with spinel LiMn_2_O_4_ as cathode in full‐cell assembly. The cathode has been chosen based on the nominal working potential (≈4 V vs. Li), high power capability, low cost and eco‐friendliness.^28^, ^29^, ^30^, ^31^ However, the Jahn–Teller distortion and poor elevated temperature performance issues cannot be neglected for the spinel cathode, but certainly it can be substantially improved by surface modification and other procedures.^29^ Apart from this, ICL is the main issue while employing α‐Fe_2_O_3_ anode in full‐cell configuration. Hence, an electrospun α‐Fe_2_O_3_ nanofiber was treated under electrochemical prelithiation process in a half‐cell configuration with Swagelok fittings for two cycles. The performance in Swagelok cell is certainly beneficial for two ways: i) overcome the huge ICL observed in the first cycle and ii) to ensure the reproducibility of the α‐Fe_2_O_3_ nanofibers observed in half‐cell assembly. Based on the electrochemical performance of both anode and cathode in half‐cell assembly under the same current density (Figure S2, Supporting Information), the mass loading between the anode to cathode ratio has been adjusted to 1:9.44. Then, the full‐cell LiMn_2_O_4_/α‐Fe_2_O_3_ nanofiber cell is cycled between 1.4–3.5 V (It is worth to mention that, the α‐Fe_2_O_3_ nanofiber anode described along with cathode is prelithiated one only). Here, the capacity is calculated based on the least mass loading of the electrode, i.e., anode α‐Fe_2_O_3_ nanofibers. The full‐cell delivered a capacity of ≈775 and ≈683 mA h g^−1^ at 0.1 C rate for first charge and discharge, respectively. Still, the irreversible capacity of ≈92 mA h g^−1^ is observed in full‐cell assembly after the pretreatment. This ICL is mainly because of the decomposition of the electrolyte, since after the pretreatment of anode, a fresh electrolyte solution has been filled during the fabrication of the cell, i.e., coin‐cell. Moreover, the small ICL observed from the cathode, LiMn_2_O_4_ cannot be ruled out (Figure S2, Supporting Information). The rate performance clearly showed the capacity profile and good capacity retention characteristics of the LiMn_2_O_4_/α‐Fe_2_O_3_ nanofiber cell. In addition, increasing current rate tends to improvement of capacity profile and coulombic efficiency well. However, at high current rate (2 C) the reversible capacity of ≈170 mA h g^−1^ only noted, i.e., slightly lower power capability than anticipated one which is mainly because of the inferior electronic conductivity profile of the hematite phase prepared. However, the power capability can be further increased by making either composite with carbonaceous materials or carbon coating. Apart from the rate performance, long‐term cycleability is another important factor for the real time applications. In this line, the LiMn_2_O_4_/α‐Fe_2_O_3_ nanofiber cell was subjected to long‐term cycleability studies at 2 C rate and the observed results are normalized and given in **Figure** **3**c. The full‐cell LiMn_2_O_4_/α‐Fe_2_O_3_ nanofiber cell displayed the retention of ≈70% of initial capacity after 4000 cycles. To date, this is the best cycleability reported on the conversion type anode in practical Li‐ion configuration. We believe such an exceptional performance of full‐cell is mainly attributed to the α‐Fe_2_O_3_ nanofiber porous morphology prepared by electrospinning, which certainly translates the better reactivity toward Li, shorter Li‐reaction pathways for the facile displacement reaction and good compatibility with the current collector. Presence of such hollow/porous structures can accommodate the volume changes reported in conversion‐based anodes, and improve long term stability. Furthermore, the appropriate optimization of the cathode, the choice of cathode and working potential cannot be neglected. The observed cycleability for LiMn_2_O_4_/α‐Fe_2_O_3_ nanofiber cell is much better than LiFePO_4_/α‐Fe_2_O_3_ configuration reported by Hassoun *et al*.,^9^ in which both cathode and anode have been pretreated before conducting the full‐cell assembly. Very recently, the same configuration is reported by Cao et al.,^27^ in which no pretreating procedure has been attempted to overcome ICL. As a result, very huge ICL is noted in the full‐cell assembly with very poor cycle life of ≈52.7% initial capacity retention after 30 cycles. Similarly, other Fe‐based oxides like magnetite phase in porous carbon matrix (54.2 wt.% carbon) showed notable cycleability in both chemically (≈64%) and electrochemically (≈58%) lithiated phase anode when paired with layered type LiNi_0.59_Co_0.16_Mn_0.25_O_2_ cathode after 1000 cycles.^8^ This clearly showed that the α‐Fe_2_O_3­_ nanofibers prepared by electrospinning displayed outstanding performance compared to the available literature and much promising for the goal of constructing high energy density Li‐ion power packs. We strongly believe that the unique one dimensional fiberous morphology, appropriate pretreating procedure, good compatibility with spinel cathode and inexpensiveness makes α‐Fe_2_O_3_ nanofibers as attractive anode for EV and HEV perspective. Further studies are in progress by fine tuning the electrode engineering, adopting carbon coating and utilizing high voltage cathode, LiNi_0.5_Mn_1.5_O_4_ to improve the cell performance for some more extend to realize the goal of high power applications.

**Figure 3:**
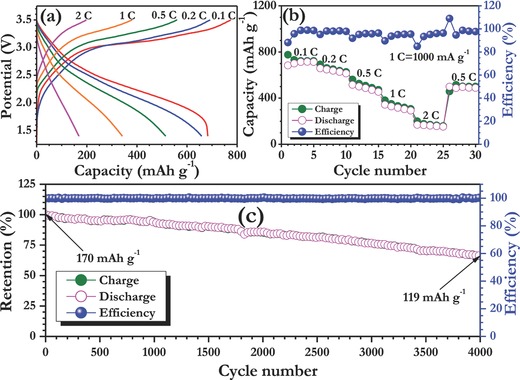
a) Typical charge–discharge curves of LiMn_2_O_4_/α‐Fe_2_O_3_ (prelithiated) cell at various current densities between 1.4 and 3.5 V, b) rate capability studies of LiMn_2_O_4_/α‐Fe_2_O_3_­ (prelithiated) cell with coloumbic efficiency, and c) long‐term cycling profiles with coloumbic efficiency. Here, 1 C is assumed to be 1000 mA g^−1^ with respect to anode loading.

To conclude, a high performance, long‐life, low‐cost, and eco‐friendly Li‐ion cell was fabricated with electrospun porous α‐Fe_2_O_3_ nanofibers as anode and spinel LiMn_2_O_4_ as cathode. An appropriate electrochemical prelithiation for anode and mass loading were conducted to attain such an exceptional performance over 4000 cycles with retention of ≈70% in full‐cell assembly. Although, the anticipated power capability was found slightly lower, but it can be certainly improved by further studies preferably adopting carbon coating or making composite with carbonaceous materials. This study certainly opens the news avenues for the development of high performance Li‐ion power packs using conversion type anode and this kind of study can be extended for rest of the conversion and alloy type anodes toward EV and HEV point of view.

## Experimental Section

Scalable electrospinning technique was used for the synthesis of porous α‐Fe_2_O_3_ nanofibers. For the spinning process, the precursors, polyvinylpyrrolidone (PVP; ${\bar{M}}_{w}$ = 1 300 000) and iron (III) acetylacetonate (Fe(acac)_3_) were purchased from Sigma‐Aldrich. Ethanol (HPLC grade) and glacial acetic acid were procured from Tedia, Singapore and used as received. First, 1 g of PVP was added in 10 mL of ethanol and stirred for 1 h for the complete dissolution at ambient temperature condition. 0.6 g of iron acetylacetonate was added to above solution and stirred continuously. Then, 1 mL of acetic acid was also added to the above solution and stirred to yield homogeneous solution. Finally, 10 mL of completely mixed precursor solutions were loaded in a plastic syringe with a hypodermic needle (dia. 22^1/2^ G). The hypodermic needle was connected to a high‐voltage supply capable of generating direct current (DC) voltages of up to 30 kV. Spinning was carried out by applying a power supply of around 17.5 kV at the needle in a controlled electrospinning set‐up (NANON (MECC, Japan)). An aluminum foil was used as the counter electrode, and the distance between the needle and the collector was fixed at 15 cm. The relative humidity level inside the electrospinning chamber was maintained around 50% during spinning. The as‐spun composite nanofiber mats were placed in a vacuum oven at room temperature for 12 h to remove the solvent residuals. Then, the electrospun polymeric fibers were calcined at 500 °C for 5 h in air at a heating rate of 5 °C min^−1^ to yield α‐Fe_2_O_3_ nanofibers.

Powder X‐ray pattern was recorded using Bruker AXS, D8 Advance diffractometer with Cu Kα radiation. The observed reflections were subjected to Rietveld refinement using Topas V3 software. Surface morphological studies were performed using field emission scanning electron microscope (FE‐SEM, JEOL JSM‐7600F) and transmission electron microscope (TEM, JEOL 2100F). CR 2016 coin‐cell assembly was used to study the electrochemical properties in standard two electrode configuration or unless otherwise stated. For the half‐cell studies, composite electrodes were formulated with 10 mg of active materials (α‐Fe_2_O_3_ or LiMn_2_O_4_, Merck KGaA, Germany), 1.5 mg of super P and 1.5 mg of teflonized acetylene black (TAB‐2) as binder using ethanol. The resultant mixture was pressed over stainless steel mesh (200 mm^2^ area with 0.25 mm thickness, Goodfellow, UK), which serves current collector. Then, the electrodes were subsequently dried at 60 °C for overnight before conducting cell assembly under Ar filled glove box. For the case of full‐cell assembly the cathode mass loading has been adjusted with respect to the anode by keeping the aforementioned conductive additive and binder ratio. The half‐cells (Li/α‐Fe_2_O_3_ and Li/LiMn_2_O_4_) were fabricated with metallic lithium as anode and composite electrodes as cathode, which was separated by microporous glass fiber separator (Whatman, Cat. No. 1825 047, UK). 1 m LiPF_6_ in ethylene carbonate (EC) and di‐methyl carbonate (DMC) (1:1 wt%, BASF) was used as electrolyte solution. Before the fabrication of the full‐cell with LiMn_2_O_4_ cathode, the α‐Fe_2_O_3_ composite electrode was subjected for two galvanostatic cycles in half‐cell assembly between 0.005–3 V vs. Li in Swagelok fittings to overcome the ICL issue. The α‐Fe_2_O_3_ composite electrode and metallic Li was separated by microporous glass fiber separator and filled with aforesaid electrolyte. After the completion of two galvanostatic cycles Swagelok fitting was dismantled and paired with LiMn_2_O_4_ cathode in the presence of new separator and fresh electrolyte in CR 2016 coin‐cell assembly with balanced mass loadings. Galvanostatic cycling profiles were recorded for both half‐cells and full‐cell assemblies using Arbin battery (2000) tester in ambient temperature conditions. Here, in both half‐cell and full‐cell assembly the capacity 1000 mA g^−1^ was assumed as 1 C.

## Supporting information

As a service to our authors and readers, this journal provides supporting information supplied by the authors. Such materials are peer reviewed and may be re‐organized for online delivery, but are not copy‐edited or typeset. Technical support issues arising from supporting information (other than missing files) should be addressed to the authors.

Supplementary

## References

[1] M. Reddy , G. Subba Rao , B. Chowdari , Chem. Rev. 2013, 113, 5364. 23548181 10.1021/cr3001884

[2] R. Satish , V. Aravindan , W. C. Ling , J. B. Goodenough , S. Madhavi , Adv. Energy Mater. 2014, 4, 1301715.

[3] V. Aravindan , J. Gnanaraj , Y.‐S. Lee , S. Madhavi , Chem. Rev. 2014, 114, 11619. 25007858 10.1021/cr5000915

[4] N.‐S. Choi , Z. Chen , S. A. Freunberger , X. Ji , Y.‐K. Sun , K. Amine , G. Yushin , L. F. Nazar , J. Cho , P. G. Bruce , Angew. Chem., Int. Ed. 2012, 51, 9994. 10.1002/anie.201201429 22965900

[5] V. Aravindan , J. Gnanaraj , Y. S. Lee , S. Madhavi , J. Mater. Chem. A 2013, 1, 3518.

[6] M. N. Obrovac , V. L. Chevrier , Chem. Rev. 2014, 114, 11444. 25399614 10.1021/cr500207g

[7] A. Varzi , D. Bresser , J. von Zamory , F. Müller , S. Passerini , Adv. Energy Mater. 2014, 4, 1400054. 10.1002/aenm.201400054 PMC4503998 26190956

[8] J. Ming , W. J. Kwak , S. J. Youn , H. Ming , J. Hassoun , Y.‐K. Sun , Energy Technol. 2014, 2, 778.

[9] J. Hassoun , F. Croce , I. Hong , B. Scrosati , Electrochem. Commun. 2011, 13, 228.

[10] R. Verrelli , B. Scrosati , Y.‐K. Sun , J. Hassoun , ACS Appl. Mater. Interfaces 2014, 6, 5206. 24611783 10.1021/am500499a

[11] R. Verrelli , J. Hassoun , A. Farkas , T. Jacob , B. Scrosati , J. Mater. Chem. A 2013, 1, 15329.

[12] P. Poizot , S. Laruelle , S. Grugeon , L. Dupont , J. M. Tarascon , Nature 2000, 407, 496. 11028997 10.1038/35035045

[13] S. Okada , J.‐I. Yamaki , J. Ind. Eng. Chem. 2004, 10, 1104.

[14] M. Biswal , A. Suryawanshi , V. Thakare , S. Jouen , B. Hannoyer , V. Aravindan , S. Madhavi , S. Ogale , J. Mater. Chem. A 2013, 1, 13932.

[15] A. Banerjee , V. Aravindan , S. Bhatnagar , D. Mhamane , S. Madhavi , S. Ogale , Nano Energy 2013, 2, 890.

[16] L. Persano , A. Camposeo , C. Tekmen , D. Pisignano , Macromol. Mater. Eng. 2013, 298, 504.

[17] V. Aravindan , J. Sundaramurthy , P. Suresh Kumar , Y.‐S. Lee , S. Ramakrishna , S. Madhavi , Chem. Commun. 2015, 51 , 2225 . 10.1039/c4cc07824a 25493289

[18] F. Badway , I. Plitz , S. Grugeon , S. Laruelle , M. Dollé , A. S. Gozdz , J.‐M. Tarascon , Electrochem. Solid‐State Lett. 2002, 5, A115.

[19] D. Shanmukaraj , S. Grugeon , S. Laruelle , G. Douglade , J.‐M. Tarascon , M. Armand , Electrochem. Commun. 2010, 12, 1344.

[20] M. W. Forney , M. J. Ganter , J. W. Staub , R. D. Ridgley , B. J. Landi , Nano Lett. 2013, 13, 4158. 23902472 10.1021/nl401776d

[21] JSR Micro, Lithium Ion Capacitor, http://www.jsrmicro.com/index.php/EnergyAndEnvironment/LithiumIonCapacitor/ (Accessed: March 2015).

[22] V. Aravindan , J. Gnanaraj , S. Madhavi , H. K. Liu , Chem. – Eur. J. 2011, 17, 14326. 22114046 10.1002/chem.201101486

[23] J. S. Gnanaraj , E. Zinigrad , L. Asraf , M. Sprecher , H. E. Gottlieb , W. Geissler , M. Schmidt , D. Aurbach , Electrochem. Commun. 2003, 5, 946.

[24] A. Debart , L. Dupont , P. Poizot , J. B. Leriche , J. M. Tarascon , J. Electrochem. Soc. 2001, 148, A1266.

[25] C. T. Cherian , J. Sundaramurthy , M. Kalaivani , P. Ragupathy , P. S. Kumar , V. Thavasi , M. V. Reddy , C. H. Sow , S. G. Mhaisalkar , S. Ramakrishna , B. V. R. Chowdari , J. Mater. Chem. 2012, 22, 12198.

[26] S. Chaudhari , M. Srinivasan , J. Mater. Chem. 2012, 22, 23049.

[27] K. Cao , L. Jiao , H. Liu , Y. Liu , Y. Wang , Z. Guo , H. Yuan , Adv. Energy Mater. 2014, 5, 14042.

[28] S. Jayaraman , V. Aravindan , P. Suresh Kumar , W. C. Ling , S. Ramakrishna , S. Madhavi , Chem. Commun. 2013, 49, 6677. 10.1039/c3cc43874k 23774756

[29] O. K. Park , Y. Cho , S. Lee , H.‐C. Yoo , H.‐K. Song , J. Cho , Energy Environ. Sci. 2011, 4, 1621.

[30] V. Aravindan , J. Sundaramurthy , P. S. Kumar , N. Shubha , W. C. Ling , S. Ramakrishna , S. Madhavi , Nanoscale 2013, 5, 10636. 24057339 10.1039/c3nr04486f

[31] S. Jayaraman , V. Aravindan , P. Suresh Kumar , W. Chui Ling , S. Ramakrishna , S. Madhavi , ACS Appl. Mater. Interfaces 2014, 6, 8660. 24766070 10.1021/am501464d

