# Supplementary material for: Ultralong Durability of Porous α‐Fe2O3 Nanofibers in Practical Li‐Ion Configuration with LiMn2O4 Cathode
Source: Adv Sci (Weinh). 2015 Mar 30;2(5):1500050. doi: 10.1002/advs.201500050 (PMC5115360; doi:10.1002/advs.201500050)
Supplement: Supplementary file 1 — Supplementary [file ADVS-2-0l-s001.pdf]

Copyright WILEY-VCH Verlag GmbH & Co. KGaA, 69469 Weinheim, Germany, 2015.

ADVANCED SCIENCE
Open Access

Supporting Information

for *Adv. Sci.*, DOI: 10.1002/advs.201500050

Ultralong Durability of Porous α-Fe_2O_3 Nanofibers in Practical Li-Ion Configuration with $LiMn_2O_4$ Cathode

Sundaramurthy Jayaraman, Vanchiappan Aravindan,* Mani Ulaganathan, Wong Chui Ling, Seeram Ramakrishna, and Srinivasan Madhavi*

Supplementary information

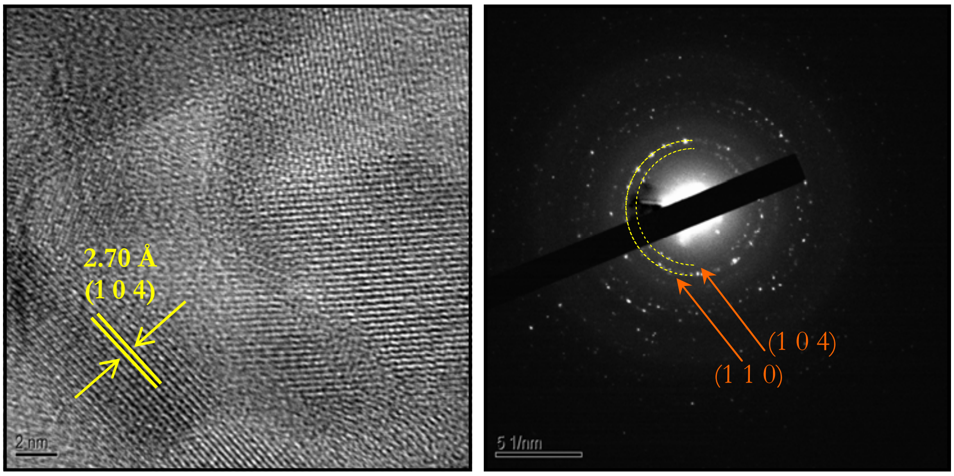

Figure S1. High resolution TEM picture and SAED pattern of porous α-Fe_2O_3 nanofibers

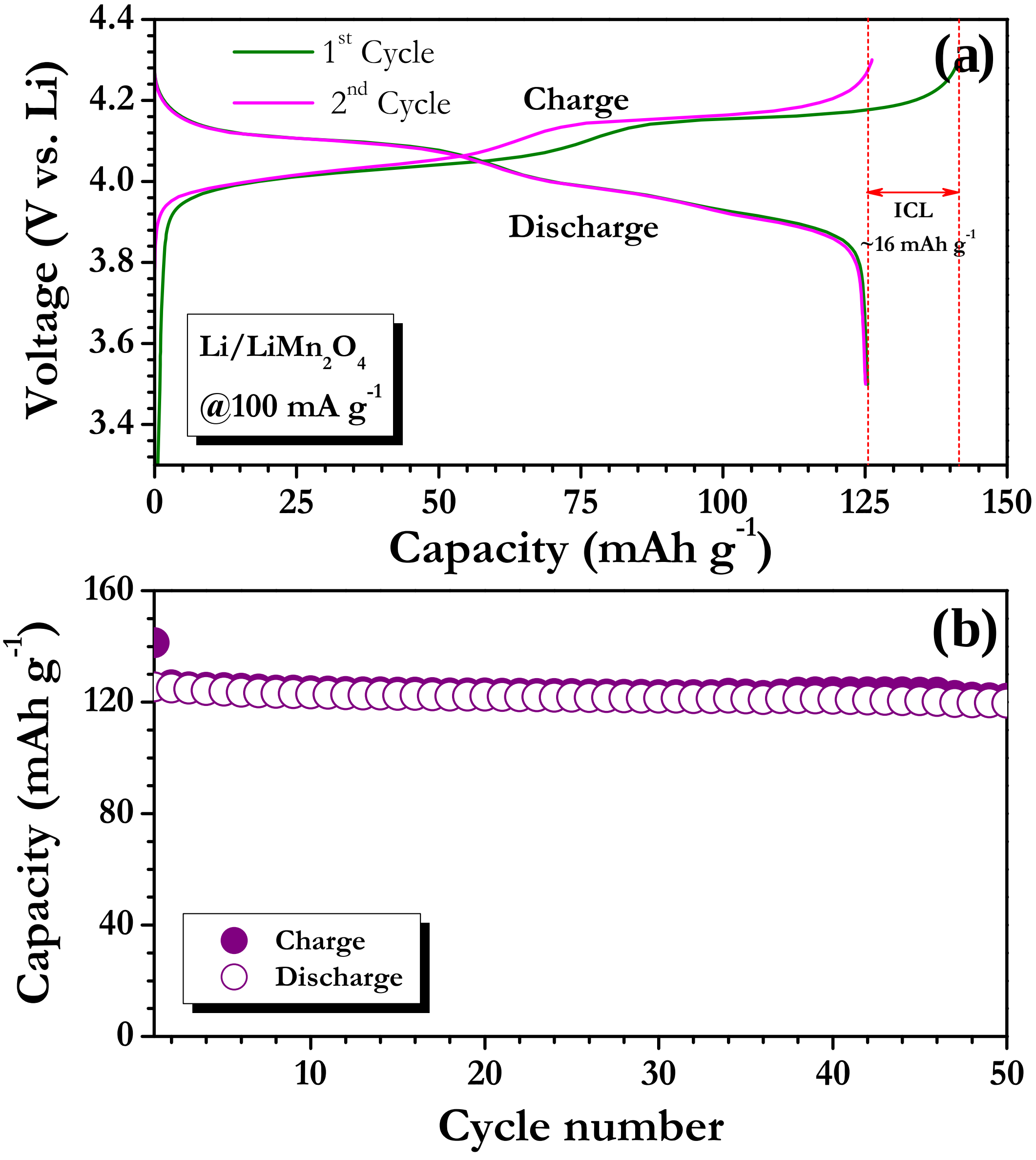

Figure S2. (a) Typical galvanostatic charge-discharge curves of Li/$LiMn_2O_4$ (Merck KGaA, Germany) at current density of 100 mA g^{-1} between 3.5-4.3 V *vs.* Li in ambient temperature conditions, and (b) Plot of capacity *vs.* cycle number
